# What Drives Consumers’ Breakfast Food Choices? Case Study in South Africa—A Multiethnic Middle-Income Country

**DOI:** 10.3390/foods15010014

**Published:** 2025-12-20

**Authors:** Colin D. Rehm, John R. N. Taylor, Henriëtte L. de Kock, Suné Donoghue, Andrew Johnson, Chanelle Thompson, Yulia Berezhnaya

**Affiliations:** 1 PepsiCo Global Research & Development, Life Sciences, PepsiCo, Purchase, NY 10577, USA; colin.rehm@pepsico.com; 2 Department of Consumer and Food Sciences, University of Pretoria, Pretoria 0002, South Africa; riette.dekock@up.ac.za (H.L.d.K.); sune.donoghue@up.ac.za (S.D.); 3 Beyond Insights, Durban 4051, South Africa; andrew@beyondinsights.co.za; 4 PepsiCo South Africa, Consumer Insights, PepsiCo, Cape Town 7530, South Africa; chanelle.thompson@pepsico.com; 5 PepsiCo Global Research & Development Life Sciences, Green Park, Reading RG2 6UW, UK; yulia.berezhnaya@pepsico.com

**Keywords:** breakfast foods, convenience, developing economy countries, food choice drivers, nutrition and health, satiety, socioeconomic factors

## Abstract

What people consume for breakfast and why they do so have not been widely studied, especially in developing-economy countries. This study aimed to determine the breakfast food habits and their drivers of adults in South Africa, a multiethnic middle-income country. An online cross-sectional survey was conducted among 1000 representative consumers of moderate to higher living standard (Living Standard Measure [LSM] range ≥ 5). Data from 842 respondents (mean age 41 years, 51.7% females and 48.3% males) was analyzed. Of 21 different food types in descending order, the most frequently consumed were bread, ready-to-eat (RTE) cereals, fruits/nuts, high-fibre cereal, yoghurt, and leftovers, all consumed weekly by 42–65% of respondents. Principal component analysis revealed that three components had eigenvalues > 1 characterized as “On-the-go”, “Traditional”, and “Ready-to-eat and functional cereals”. They explained 49% of the data. Decision tree analysis revealed that, for example, Black respondents were more likely to consume foods in the “traditional” category. Quick-and-easy options, notably bread, RTE cereals, fruits/nuts, and leftovers, were dominant, especially among lower LSM respondents. Tasty and filling, and value for money, as exemplified by leftovers and vetkoek (fried dough), were important considerations, particularly among these respondents. These drivers can lead to unhealthy choices, a major concern in South Africa with its high level of diet-related diseases. This study, however, indicates that South African consumers, irrespective of age, ethnicity, and living standard, rated healthfulness and nutritional value highly as a benefit, the highest for choosing 13 of the 21 foods. Additionally, aspects of wellness, e.g., feeling energized/recharged, rated very highly. Thus, it is concluded that the opportunity exists to support consumer needs of nutrition and wellness together with affordability, taste, and satiety.

## 1. Introduction

Breakfast, literally break or end of fasting, is the first meal of the day and is usually consumed before work, school or other daily activities. It is often referred to as “the most important meal of the day” [1,2]. There is considerable evidence that consuming breakfast, as opposed to not consuming breakfast, confers several nutritional and health benefits. These benefits include a higher micronutrient intake [3], higher nutrient adequacy ratio and Healthy Eating Index score [4,5], more stable glycemia [6], better insulin sensitivity [7], and control of energy intake [8]. There is also evidence that consumption of breakfast improves mental well-being [9], can improve memory in adults [10], and possibly aids in specific aspects of cognitive function in adolescents and children [11].

Food choice is generally considered as having four primary drivers, namely taste, cost, convenience, and perceived health/nutritional value [12]. With specific reference to South Africans, seven factors have been identified that influenced food choices, namely healthy eating constraints, frugality, emotional eating, meat appeal, the weather, quality seeking, and cooking constraints [13]. Given the important contributions of breakfast to nutrition, health, and mental well-being, a key issue is what factors drive consumers’ breakfast food choices. Surprisingly, there has been little research on this subject. Studies in Europe [14] and the USA [15] on the breakfast habits of adolescents revealed, for example, that adolescents in families with low socioeconomic status were more likely to consume breakfast of low nutritional quality than their peers. Further, eating breakfast together as a family can benefit adolescents’ dietary intake and weight status, but the mechanisms driving this association are unclear. A consumer survey of more than 1000 adults in the UK found that the major reasons stated for consuming breakfast were provision of energy, alleviation or prevention of hunger, and enjoyment [16]. This was reflected to an extent in differences in the choice of foods consumed during weekdays and weekends [16]. The most common weekday breakfast foods were cereals, bread or toast, and porridge (presumably oats/muesli). For weekends, there was a considerable increase in the consumption of eggs, bacon and sausages, and cooked (English) breakfast. A survey of 460 residents of Switzerland found that attention to healthy food choices, higher education attainment, being female, and higher reported fitness levels were linked to consuming “healthier” breakfasts [17].

The vast majority of breakfast consumption research has concerned consumers in higher-income countries in Europe and North America. Research on consumers’ breakfast habits in developing economy countries is lacking. South Africa, dubbed the “Rainbow Nation” due to its population diversity, is on average a middle-income country of 62 million people (2023 figure) but with a very wide disparity of wealth. The population consists of 81.0% Black Africans, 8.7% Coloureds (mainly mixed race), 7.8% Whites of European descent, and 2.6% people of Indian descent. The population is predominantly urbanized (69%) and is well along the trajectory of nutrition transition from a traditional rural African diet to a Western-style diet [18,19]. The very high incidence of obesity and associated lifestyle-related diseases reflect this shift [20]. These characteristics make South Africa an ideal setting to conduct research on drivers of food intake outside of Europe and North America.

There is some South African-based research on breakfast consumption versus not consuming breakfast and its potential impact on nutrition and health. Most of these studies were conducted in relatively finite and selected populations such as school children/adolescents, college students, and healthcare professionals. There are several studies of children and adolescents, due largely to the importance of school meals in addressing undernutrition [21,22,23,24,25,26,27]. Overall, the studies found some evidence of positive impacts of breakfast consumption on learners’ nutritional status and school attendance, but evidence of effects on education performance was more circumstantial. A cross-national study of more than 21,000 college students from 12 countries found that students in Cape Town, South Africa, had amongst the lowest percent reporting frequent breakfast consumption (65.6% versus 81.2% in the total sample) [28]. A study of healthcare professionals observed breakfast to be the most skipped meal and that the incidence of consuming breakfast increased with age [24].

As indicated, few breakfast food consumption studies have been conducted in developing-economy countries, and with specific reference to South Africa there have been few among adults. More importantly, no published studies have attempted to capture breakfast food consumption habits in the general adult population. This paper aims to help fill these gaps in knowledge. It describes the findings of an online survey to determine the breakfast foods consumed by South African adults of moderate to higher living standard and the factors that drive their breakfast habits. These population segments were selected as they are likely to have adequate income to make a broad range of food choices. It is hypothesized that a wide range of breakfast foods will be consumed, given that, as described, South Africa is in the midst of a nutrition transition and, further, that the factors driving South Africans’ breakfast foods habits will be complex given the diversity of the population and living standards of the population.

## 2. Materials and Methods

### 2.1. Study Design and Implementation

A cross-sectional quantitative online survey of 1000 adults of approximately equal numbers of females and males, approximately in proportion to the South African population groups (races) aged 18–64 years (y) of South African Living Standards Measure (LSM) levels 5–10 (defined in Section 2.2 below) residing in all areas of South Africa, was performed in December 2022. The questionnaire consisted of 26 questions concerning three different but interrelated topics: respondents’ breakfast consumption habits and attitudes towards breakfast (12 questions); their whole-grain knowledge, opinions, and health benefits awareness (9 questions); and their self-reported health and attitudes regarding health and wellness (4 questions). Previously, the present authors used particular sections of the questionnaire to report on the respondents’ attitudes, knowledge, and behaviours towards whole grains [29]. The questionnaire comprised structured, closed-ended questions, including multiple choice (single- or multiple-response), ranking, and rating scale questions. The rating scale questions used Likert-type formats, including a 9-point scale ranging from strongly disagree to strongly agree, a 5-point scale ranging from poor to excellent, and a 5-point scale ranging from not at all concerned to very concerned.

A list of 21 breakfast food types was presented to the respondents in random order. Because South Africa’s British colonial past has strongly influenced the types of breakfast foods consumed [30], the selection of breakfast food types was loosely based on those used in a questionnaire-type study of UK consumers [16]. Through two consumer focus discussion groups, each comprising eight people generally representative of adult South Africans, the list was expanded to include breakfast foods particular to the Southern Africa region. The types were further selected to distinguish between convenience-type quick-to-prepare, and traditionally cooked cereal foods and to include more recently available breakfast foods such as functional cereals, shakes, and drinking yoghurt. Because the study specifically concerned foods, beverages such as tea, coffee, fruit juices, and soft drinks were excluded.

The questions were phrased using terms that South African consumers would understand. Validation of the instrument involved refining the draft questionnaire through the two consumer focus discussion groups. Following this, the wording of the questions was reviewed and amended by the panel provider and the authors. For example, additional colloquial names and examples of actual brand names were provided for several food types to facilitate respondents’ understanding. Demographic and sociodemographic questions (age group, gender, population group (race), province of residence, presence of children in the household, respondent living standard—defined in detail below) and selected health behaviour questions (self-reported health status, self-reported use of any special diets in the past year, and attitudes toward breakfast) were included.

Respondents had to indicate how often they consumed the 21 breakfast foods per week. Responses were then dichotomized into defining regular consumers of each food based on reporting consuming them ≥2–3 times per week. To obtain more detailed information on the context of consuming a given breakfast food, regular consumers of such foods had to answer follow-up questions about just three foods to limit respondent fatigue. These questions assessed satisfaction, when the food was consumed, where the food was consumed, with whom it was consumed, and how soon after waking it was consumed. About 15% of respondents were randomly selected to answer these detailed questions. The responses per food represented subsamples of the data, which could potentially overlap with one another. These respondent subsamples were also asked as to how they wanted to feel when they consumed a given breakfast food, e.g., “to have a fresh start” or “to identify with my culture/tradition” and what they wanted to obtain from each breakfast food, e.g., “is tasty”, “provides the best for my family”, “helps with digestion”.

The actual questions and response options relevant to the present research are provided in the Supplementary Materials.

### 2.2. Survey Administration and Sampling

Ethical approval for the study was obtained from the University of Pretoria’s Ethics Committee (Ethics Number NAS357/2022). Respondents provided informed consent to participate in the study voluntarily and anonymously. They could withdraw from the study at any time. Respondents completing the questionnaire received a 30 South African Rands store voucher as an incentive for participating.

Respondents were recruited by Borderless Access (a market research company) through its database of some 300,000 South Africans. Panellists who met the criteria for participation were invited via e-mail. They provided informed consent by clicking on a link after reviewing the study synopsis, research procedure, and their rights as participants.

Respondents were pre-screened to ensure quotas for gender, population group (race), age group, and living standard, i.e., socioeconomic status. Living standard was assessed using the South African Living Standards Measure (LSM). This ranges from 1 to 10 and is based on access to basic amenities (e.g., tap water or hot running water in the dwelling) and the ownership of certain durable goods (e.g., electric stove or more than one cell phone in the household) [31], of which any one of these would qualify as LSM 5. Adults of LSM levels 5–10 were selected on the basis that these persons are likely to have adequate disposable income to be able to make a broad range of food purchase choices. Data were collected over ten days from equal numbers of females and males in approximate proportion to South Africa’s population groups. Only individuals reporting that they consumed breakfast 2–3 times a week or more were eligible to participate.

### 2.3. Analysis Approach

Before initiating formal analysis, it was determined that several individuals reported consuming an implausibly high number of breakfast food types in a week. Respondents who reported consuming five or more of the 21 breakfast food types every day or nearly every day were therefore excluded from the data analyses. These respondents accounted for 15.8% of the total; consequently, data from 842 respondents were analyzed. Excluding reported daily or nearly daily consumption of more than four foods aligns with published findings on breakfast food consumption patterns. A survey of UK consumers revealed that four different food types were commonly consumed (>25% of respondents) by adults in the middle socioeconomic tertile, and six foods somewhat less commonly (>20%) [32]. Similarly, in the USA, four different breakfast food types were found to be the most commonly consumed by adults (>15% of consumers) [33].

### 2.4. Principal Component Analysis (PCA), Univariate ANOVA, and Decision Tree Statistical Analyses

PCA was applied to identify similarities in the frequency of consuming the breakfast food types. The first model included all 21 food types. Cross-loaded types and those with communalities < 0.30 were excluded after the first and second iterations. The parallel analysis method determined retention of three principal components (PCs) (breakfast food categories), 1, 2, and 3. As the three components all had eigenvalues greater than 1 when parallel analysis was applied, it is considered that the model has validity. Parallel analysis was applied to assess validity as it compares the observed eigenvalues with those obtained from randomly generated data of the same size and structure [34]. The Promax rotation was used as the components were correlated.

Three Univariate ANOVAs were run to model the effect of the pre-specified independent variables, i.e., age, gender, population group, living standard (LSM), perceived health, concern about health conditions (14 types), and breakfast attitude (7 types), on three dependent variables, the principal components 1 (on the go), 2 (traditional), and 3 (RTE and functional cereals). The final Univariate ANOVAs report only the significant independent variables as follows:

PC1: On-the-go = μ + β_1_(Population group) + β_2_(Gender) + β_3_(LSM) + β_4_(Perceived Health) + β_5_(Hypertension Concern) + β_6_(Taste Importance) + β_7_(Whole Grain Seeking) + ε

PC2: Traditional = μ + β_1_(Age group) + β_2_(Population group) + β_3_(LSM) + β_4_(Perceived Health)_4_ + β_5_(Hypertension Concern) + ε

PC3: RTE and functional cereals = μ + β_1_(Population group) + β_2_(LSM) + β_3_(Perceived Health) +β_4_(Whole Grain Seeking) + ε

where μ is the intercept, β is the beta coefficients, the predictor variables are in brackets, and ε is the random error term or residual, where the F values for the ANOVA model are as follows: F = MS_effect_/MS_error_.

The 5-point scale response options for self-reported health were merged into fewer categories (3-point scale): poor to fair health = 1, good health = 2, and very good to excellent health = 3. Similarly, the response options for concern about health conditions were combined into not concerned = 1, neutral = 2, and concerned = 3. The 9-point agreement scale for attitude to particular food types (seven types with wording about breakfast choices or consumption) was treated as a continuous measure.

The final Univariate ANOVAs report only the significant independent variables. In each case, Levene’s Test for Equality of Error Variances verified that the variances were equal across groups or samples, with *p*-values > 0.05. Bonferroni post hoc tests (pairwise comparisons) were performed after finding statistically significant results to determine group differences.

The covariates, continuous variables controlled for when examining the effect of an independent variable on a dependent variable, were evaluated at the following mean values: “Taste is more important in breakfast than health and wellness” (mean = 4.87) and “I actively look for whole grain foods for my breakfast occasions” (mean = 6.09).

Next, decision tree analysis was performed to further explore the relationship between the significant independent variables and consumption frequency per breakfast food category. The decision trees illustrate the characteristics of respondents that influence consumption frequency per breakfast food category, as well as the specific sub-groups of respondents who frequently consume within the category versus those who do not.

PCA and decision tree analyses were conducted using JASP software (0.18.3, 2024).

### 2.5. Heatmaps: Summarizing Patterns and Determinants of Individual Food Choices and Statistical Analysis

Heatmaps were compiled to visually evaluate patterns of breakfast food type consumption and drivers of breakfast food type consumption across the entire assessed population and by specific sociodemographic or subsample defined by age group, gender, LSM, population group (race), presence of children in the household, self-reported health status, and reported use of special diets in the past year. A chi-squared test was used to determine if intake of a given breakfast food type differed across these population sub-groups. To aid interpretation, the breakfast food types were visually grouped in a manner consistent with the PCA results. Additionally, to facilitate reporting of the results, one-word short-hand names for each breakfast food type were identified. On the first use of each term, the full-name and short-hand names are used, and only the short-hand name is used thereafter.

For health concerns and general attitudinal questions towards breakfast and general wellness, generalized linear models were employed using a log-link and binomial distribution to estimate the prevalence ratio of food consumption for individuals reporting a given health concern or strongly agreeing with each attitudinal question [35].

Data management and standard statistical analyses were conducted in Stata 17.0 for Windows (College Station, TX, USA) and SPSS for Windows, Version 26 (Armonk, NY, USA). *p*-values less than 0.05 were considered statistically significant.

## 3. Results

### 3.1. Respondent Characteristics

The largest age group were 25–34 y, just over 30% of the respondents, and there was roughly an equal number of males and females (Table 1). About 60% of the respondent sample had an LSM level of 5–7 (moderate socioeconomic status), and most respondents considered themselves Black. More than three-quarters of the sample reported having children living in their household. Most respondents reported being of very good/excellent or good health status. Across the sample, 72% of respondents reported consuming breakfast daily. There were some differences in the prevalence of daily breakfast consumption, with older participants and individuals with LSM levels of 5–7 being more likely to report consuming breakfast daily. Black participants also reported being daily breakfast consumers more often than the other population groups. Having children in the household was not associated with daily breakfast consumption. People with better self-reported health were more likely to report daily breakfast consumption.

### 3.2. PCA Results, Univariate ANOVAs and Decision Trees for Each Principal Component

**Figure 1:**
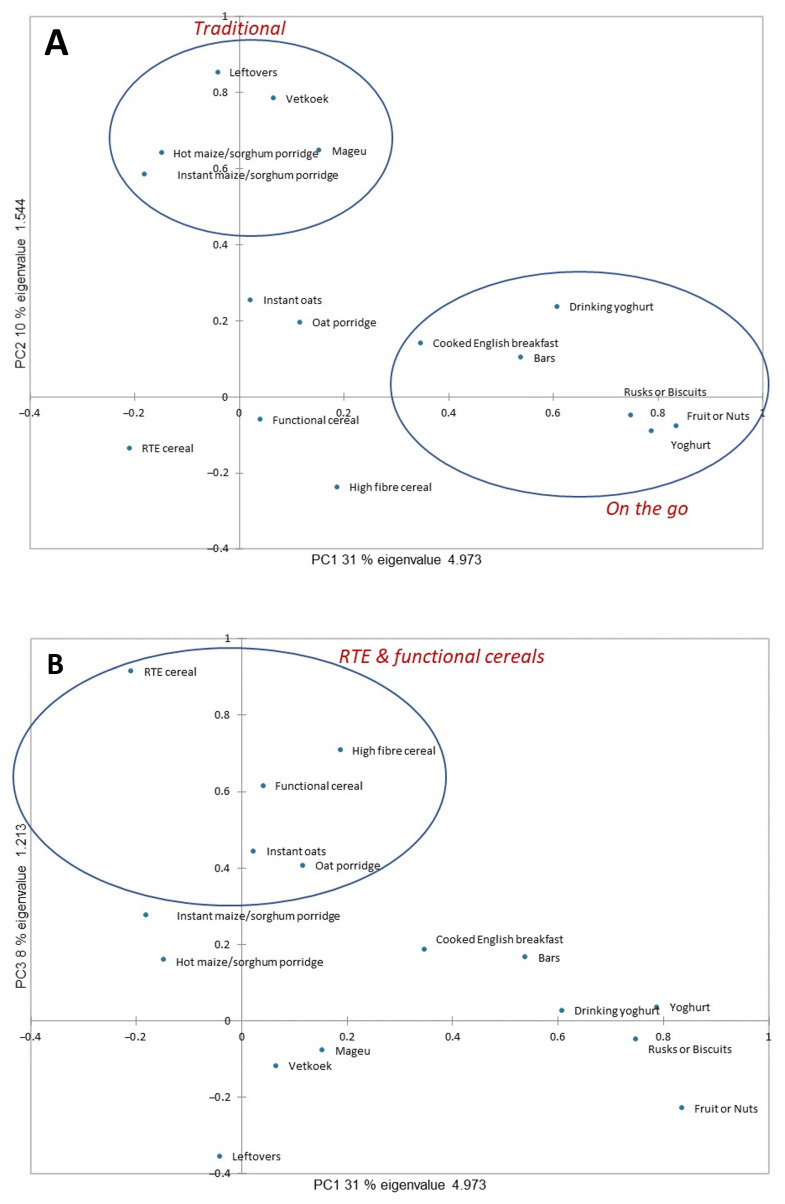
Plots of the optimized principal component analysis model depicting associations of 16 of the breakfast food types based on similarity in consumption frequency. (**A**) PC1 versus PC2, (**B**) PC1 versus PC3.

The optimized PCA model identified three principal components (PCs) (breakfast food categories) (Figure 1) all with eigenvalues >1.0, as determined by parallel analysis, with eigenvalues >1 being generally considered as significant [34]. They explained 49% of the variation in consumption of breakfast food types. In social sciences research such as this study where the information obtained is not precise, a solution which accounts for 60% of the total variance is generally considered acceptable, and in some instances a lower percentage is acceptable [36]. Not all breakfast foods were included in the components. Bread/toast/sandwiches (bread) were excluded because their intake across the entire population dominated the analysis. Breakfast shakes/protein shakes (shakes), pastries/pancakes/waffles (pastries/pancakes), muesli, and cream of maize/cream of wheat (cream of maize/wheat) were excluded due to cross-loading or low communality.

PC1 explained 31% of the variance and comprised fruits/dried fruits/nuts (fruits or nuts), yoghurt, rusks/breakfast biscuits/savoury biscuits (rusks/biscuits), drinking yoghurt, energy/cereal/breakfast bars (bars), and cooked English breakfast (eggs/bacon/sausage) (cooked English breakfast) (Figure 1A). This component was subsequently categorized as the “On-the-go” food category. PC2 explained an additional 10% and was categorized as the “Traditional” food category and comprised leftover food (leftovers), amagwinya/vetkoek (vetkoek), hot porridge (e.g., maize porridge, mabele, and maltabella), (hot maize/sorghum porridge), mageu/motoho (mageu), and instant hot porridges (just add water, e.g., instant maize, mabele, etc.) (instant maize/sorghum porridge). Lastly, PC3 added 8% to the explanation and was categorized as the “RTE and functional cereals” food category and comprised ready-to-eat cereal (RTE cereal), high-fibre cereal and functional cereal, cooking oats (oat porridge), and instant oats/just add hot water oats (instant oats) (Figure 1B). Thus, most of the food items themselves and the grouped food categories were diverse, leading to lower shared variance among indicators. The 49% variance explained reflects this inherent “noise” in the measurements but is a reasonable result in this context.

Univariate ANOVA 1 revealed significant effects of population group (*p* = 0.001), LSM (*p* = 0.015), perceived health status (*p* < 0.001), concern about hypertension and blood pressure (*p* < 0.001), agreement with taste is more important in breakfast than health and wellness (*p* < 0.001), and agreement with “I actively look for whole grain foods for my breakfast occasions” (*p* < 0.001) on the consumption frequency of foods in the “On-the go” food category (Table 2). The Black population group consumed foods from the “On-the-go” category significantly more frequently than the Coloured/Indian and White groups (*p* < 0.001). Univariate ANOVA 2 (Table 2) showed that age (*p* = 0.003), population group (*p* < 0.001), LSM (*p* = 0.007), perceived health status (*p* < 0.001), and concern about hypertension and blood pressure (*p* < 0.001) all had a significant effect on the consumption frequency of foods from the “Traditional” category. Univariate ANOVA 3 (Table 2) revealed significant effects of population group (*p* = 0.002), LSM (*p* < 0.001), perceived health status (*p* < 0.001), and agreement with “I actively look for whole grain foods for my breakfast occasions” (*p* < 0.001) on the “RTE and functional cereals” category.

For the reasons explained above, bread, shakes, pastries/pancakes, muesli, and cream of maize/wheat were excluded from this optimized model.

**Figure 2:**
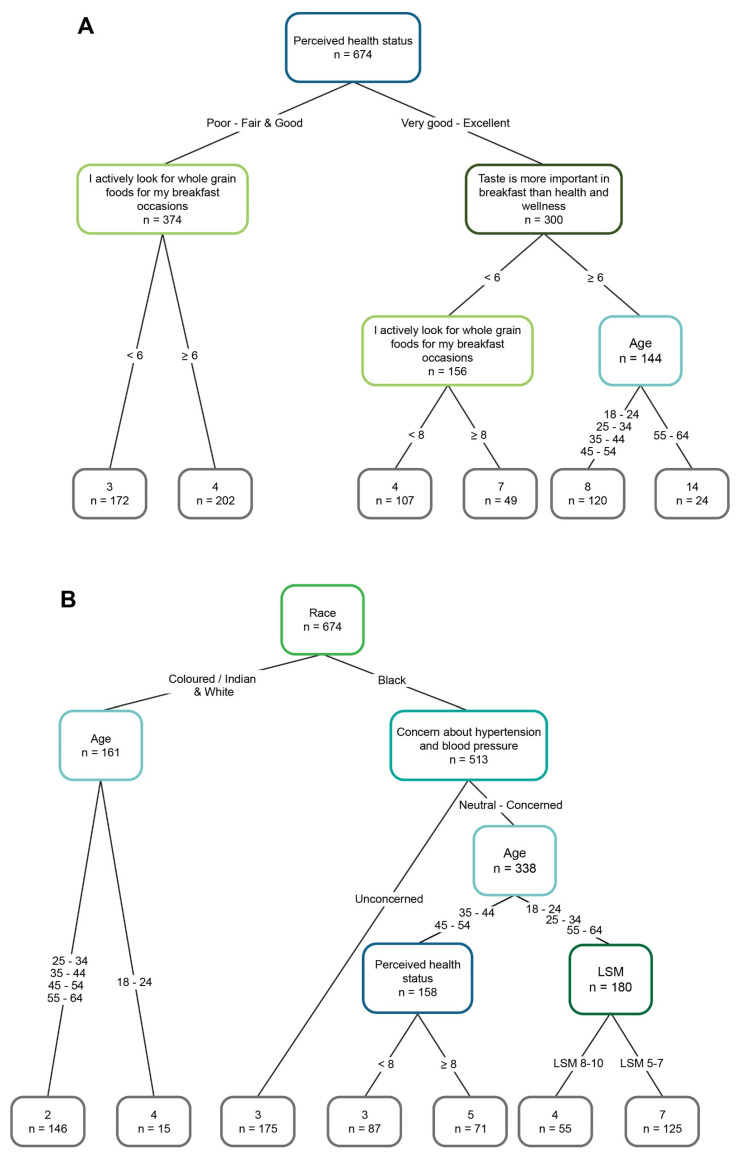

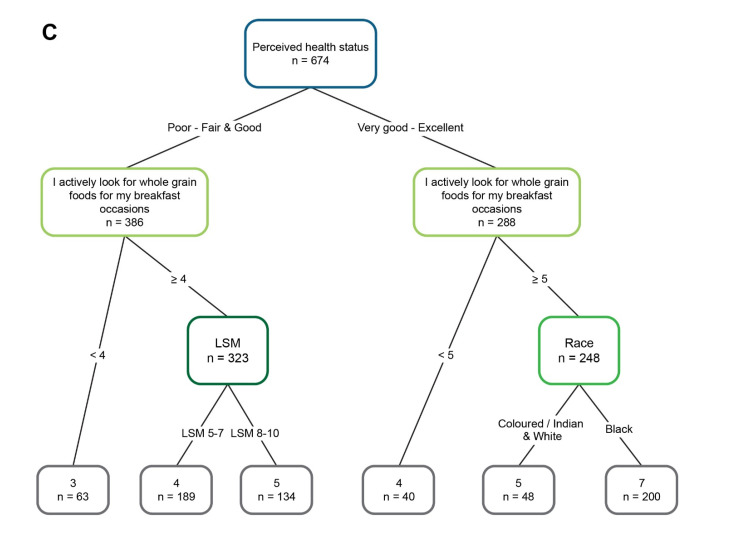
Decision trees showing the effects of variables associated with the consumption frequency of (**A**) “On-the-go”, (**B**) “Traditional”, and (**C**) “RTE and functional cereals” food categories.

Decision tree analysis showed that self-reported health status was the primary differentiating factor for the consumption frequency of the “On-the-go” category (Figure 2A). Respondents with poor to fair and good self-reported health status and actively looking for whole-grain breakfast foods were inclined to consume the “On-the-go” category foods more frequently than those less likely to search for whole-grain foods. For those with very good to excellent health status, the attitude to food type and the taste of food was more important than health and wellness further differentiated the consumption frequency factor. Respondents aged 55–64 consumed within this category more frequently.

Population group (race) had the strongest association with the consumption frequency of the “Traditional” food category (Figure 2B). Various factors were related to Black respondents’ consumption frequency of this category, specifically concern about hypertension and blood pressure, age, LSM, and perceived health status, which are indicative of more complex decision-making. In contrast, the consumption frequency of the “Traditional” category by Coloured/Indian and White respondents differed only according to age group. The consumption frequency was higher for Coloured/Indian and White respondents between 18 and 24 y than those 25–64 y. Coloured/Indian and White consumers generally consumed less traditional African foods and instead consumed predominantly Western-type foods.

Perceived health status had the strongest association with the consumption frequency of the “RTE and functional cereals” food category (Figure 2C). Consumption frequency for this category was higher for those agreeing strongly that they actively searched for whole grains for breakfast. In the very good to excellent health status group, Black respondents were more likely to consume within the category than Coloured/Indian and White respondents. For the poor to fair and good health status group, differentiation based on living standard determined consumption frequency.

The sample comprised 674 respondents (80% of the total sample (n = 842), and 20% were used as a holdout test sample.

### 3.3. Heatmaps and Consumption Patterns for Individual Foods

**Figure 3:**
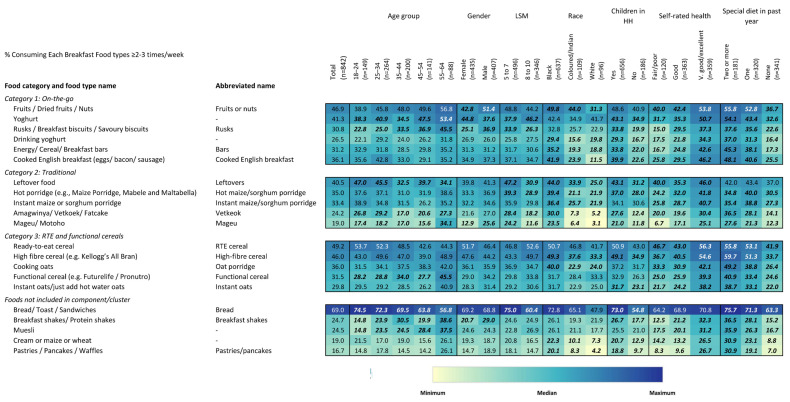
Heatmap of consumption of each breakfast food type overall and by sociodemographic strata. Values are the percentage of respondents who reported consuming a particular food type ≥ 2–3 times a week. Values in bold and italics are statistically significant at the 0.05 level. Colours correspond to size of values corresponding to colours in the legend. Colours are coded for each question separately.

The descriptive heat maps summarize sociodemographic, context of consumption, and potential drivers of consumption in terms of emotional and functional drivers of consuming the individual breakfast foods types. Across the entire respondent sample, bread, RTE cereal, and fruits or nuts were the most consumed breakfast foods (Figure 3). Sociodemographic and behavioural factors, including age, gender, LSM, population group, presence of children in the household, and reported use of any special diets in the past year, were all related to the types of breakfast foods reported. Some foods were more commonly consumed by older individuals, including muesli, rusks/biscuits, and shakes, while a limited number were preferentially consumed by younger individuals, including leftovers and bread (*p* < 0.05 for all reported comparisons). Only a few breakfast foods were consumed differentially by gender, with shakes, fruits or nuts, mageu, and rusks/biscuits being more reported by men. Yoghurt was more commonly reported by women (*p* < 0.05).

Among respondents of the lower LSM level (5–7), vetkoek, mageu, and leftovers were preferentially consumed, while higher LSM individuals (8–10) preferentially consumed yoghurt (Figure 3). For most breakfast foods, Black respondents tended to be more frequent consumers, a trend that was especially strong for vetkoek, mageu, pastries/pancakes, and cream of maize/wheat. Respondents with children in the household were also more likely to consume nearly all assessed food categories, especially vetkoek, cooked English breakfast, pastries/pancakes, and drinking yoghurt (*p* < 0.05). With respect to health status, individuals reporting excellent health were more likely to consume most breakfast foods, with an especially strong trend for mageu, pastries/pancakes, shakes, bars, and rusks/biscuits. Hot maize/sorghum porridge and pastries/pancakes were both much more likely to be reported by individuals who reported consuming a special diet in the past year (*p* < 0.05).

**Figure 4:**
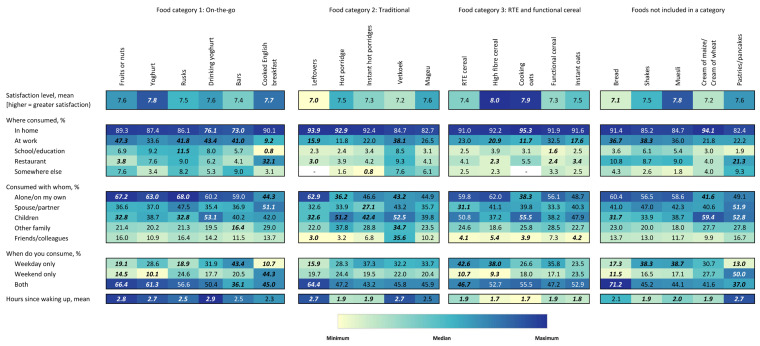
Heatmap of satisfaction level and context of consumption for each food type. Values are the percentage of respondents who reported consuming the food type in the given context. Values in bold and italics are statistically significant at the 0.05 level. Colours correspond to size of values corresponding to colours in the legend. Colours are coded for each question separately.

Satisfaction level and context of consuming specific breakfast foods are shown in Figure 4. Respondent satisfaction levels regarding the breakfast foods were generally very high, above 7 (out of a scale of 9) for all assessed foods. Satisfaction levels were lowest for leftovers and highest for high-fibre cereal and oat porridge. Across most assessed food types, home was the most common location of consumption, distantly followed by work. Restaurants were important places for a few foods, namely cooked English breakfast and pastries/pancakes. Some foods were more often consumed with children (e.g., oat porridge, maize/sorghum porridge) versus other foods being consumed alone (e.g., bread, shakes, bars, fruits or nuts, leftovers, and rusks/biscuits). Vetkoek was consumed at higher levels with friends/colleagues. Cooked English breakfast and pastries/pancakes were more often consumed on weekends. Lastly, some foods were consumed sooner after awakening than others, notably functional cereals, high-fibre cereal, oat porridge, instant oat porridge, and RTE cereal. Yoghurt, drinking yoghurt, and fruits or nuts were consumed later.

**Figure 5:**
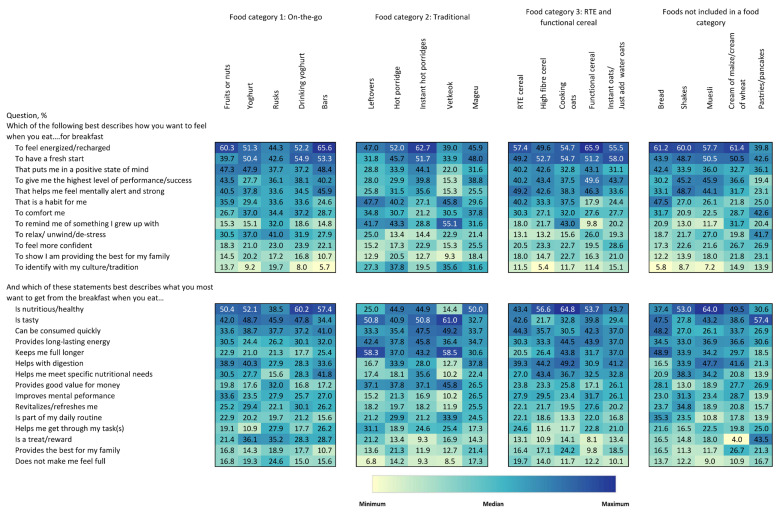
Heatmap of emotional and functional benefits for consuming each food type among a randomly selected subset of participants consuming the food type. Values are the percentage of respondents who selected the statements which best described how they wanted to feel or what they wanted to obtain from consuming a particular food type. Values in bold and italics are statistically significant at the 0.05 level. Colours correspond to size of values corresponding to colours in the legend. Colours are coded for each question separately.

Figure 5 shows the emotional and functional benefits being sought. In terms of emotional benefits, “to feel energized/recharged”, “to have a fresh start”, and “that puts me in a positive state of mind” were the three highest-ranking responses across all food types, though considerable heterogeneity was observed. “This is a habit for me” ranked highly for vetkoek, bread, oat porridge, and leftovers. “To remind me of something I grew up with” ranked highly for vetkoek, oat porridge, and leftovers, as did “To identify with my culture/tradition.” When considering functional benefits, “is nutritious/healthy” followed by “is tasty” and “can be consumed quickly” were the top-ranking average responses across all breakfast foods. Nutrition appeared to be very important for oat porridge, drinking yoghurt, and muesli, with more than 60% of respondents flagging this attribute. “Is tasty” stood out for vetkoek, leftovers, pancakes, and instant oat porridge. “Can be consumed quickly” was a leading factor for vetkoek, bread, instant oat porridge, and RTE cereal. Other notable pairs of breakfast food types and benefits included vetkoek for “provides good value for money” and “keeps me full longer”, pastries/pancakes for “is a treat/reward”, leftovers for “keeps me full longer”, and muesli for “helps with digestion”.

**Figure 6:**
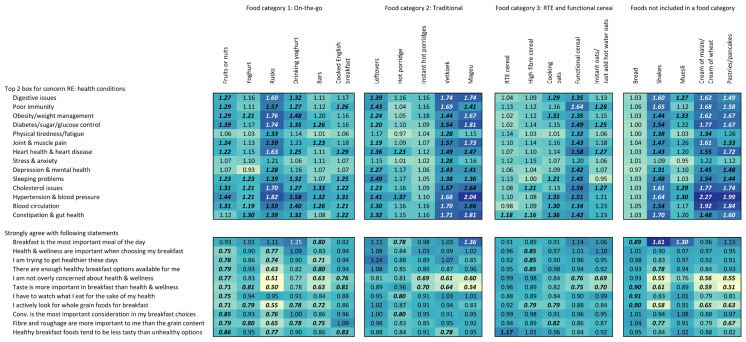
Heatmap of association (prevalence ratio) between reported health concerns and agreement with attitudinal questions regarding breakfast and general wellness and consumption of each food type. Values can be interpreted as the prevalence ratio or, more specifically, the ratio of the proportion of individuals reporting consumption of each food who report each health condition or agree with the statements versus those who did not have each health condition/agree with each statement. For example, the value of 1.61 for breakfast shakes and “Breakfast is the most important meal of the day” means that individuals indicating that they agree with that statement are 61% more likely to report consuming shakes for breakfast than individuals who do not agree with that statement. Values in bold and italics are statistically significant at the 0.05 level. Colours correspond to size of values corresponding to colours in the legend. Colours are coded for each question separately.

Figure 6 shows the association between consumption of each food type and the self-reported concern for specific health issues and with strongly agreeing with specific statements regarding health, nutrition, and general wellness. Consuming rusks/biscuits was associated with high levels of concern for numerous health issues, including digestive issues, obesity/weight management, diabetes, heart health, cholesterol issues, and hypertension. Vetkoek and mageu consumption were also associated with increased likelihood of reporting concern for numerous health outcomes, including hypertension, constipation and gut health, poor immunity, and digestive issues. Functional cereal, shakes, cream of maize/wheat, and pastries/pancakes were also associated with concern for numerous health outcomes. An increased likelihood of strong agreement with the statement “Breakfast is the most important meal of the day” was associated with consuming shakes and muesli. This association may be related to these consumers’ relatively high ratings for wanting to feel energized/recharged, having a fresh start, reach one’s highest performance, and feel mentally alert when they consume these perceived “healthy-type” breakfast foods. Consumption of rusks/biscuits, vetkoek, and mageu was associated with decreased likelihood of strongly agreeing to the statements “Taste is more important in breakfast than health and wellness” and “I am not overly concerned about health and wellness”.

## 4. Discussion

This is the first study that provides detailed information on the breakfast consumption patterns of a significant segment of the South African adult population. As described in the Introduction, previous studies on breakfast consumption in South Africa have focussed on the impact of consuming and not consuming breakfast on nutrition and health, with most being conducted on specific populations. Drivers of breakfast food choice in South Africa are clearly complex. This present study indicates that all factors determined by Dlamini et al. [13], except for weather, which was not assessed, were considerations in the respondents’ choice of breakfast foods. Moreover, specific social and cultural considerations clearly also came into play (see the deep dives below). Although the breakfast food types were predominantly consumed at home (>80% consumption), their consumption together with children was much lower, between 30 and 60%. This is in general agreement with a survey of primary school learners in a low-to middle income district of Cape Town, South Africa, which revealed that although most households had rules about consuming unhealthy beverages and snacks, breakfast was only consumed as a family less than two times a week [37].

Concerning perceived benefits of consuming the various breakfast food types, healthfulness and nutritional value were amongst the most reported benefit areas and were the top reported benefit area for 13 of the 21 breakfast food types. Taste was the top perceived benefit area for 5 of the 21 types, namely rusks, leftovers, instant porridges, vetkoek, and pastries/pancakes. Convenience was rated highly, but it was not the top benefit statement for any of the food types. The related benefit of “can be consumed quickly” was also rated highly for several of the food types and was the top benefit associated with RTE cereal.

While South African consumers are, by necessity, very cost-conscious [38], among this sample of moderate and high socioeconomic status people, food types providing good value for money did not rank near the top for most foods. This raises the possibility that individuals may assess/rank breakfast food types differently than other aspects of their diet, placing a somewhat more important role on health/nutrition and other perceived benefits (e.g., digestion, long-lasting energy) than cost alone.

Probably by necessity (i.e., long commuting times, many single-parent families, lack of cooking facilities), quick-and-easy breakfast food options, notably bread, RTE cereals, fruits and nuts, and leftovers, were dominant among consumers, especially among respondents of lower LSM. Tasty and filling foods, e.g., vetkoek and leftovers, clearly drove many breakfast decisions. Value for money, for example, in the case of vetkoek, was a major concern, especially for those on tight budgets. In a survey of South African consumers for food choices, “healthy eating constraints” was the most prominent predictor of food choice, irrespective of their living standard [39]. Unfortunately, these drivers can lead to unhealthy choices, particularly in developing-economy countries, which have high rates of diet-related diseases.

### 4.1. Deep Dives into Specific Components

As would be expected, there was considerable heterogeneity in the drivers of consumption of the individual food types versus the broader food categories. To better unpack these drivers, deep dives into some particular food types and the associations revealed by PCA are provided.

#### 4.1.1. Bread Deep Dive—Vetkoek Compared to Conventional Bread

PCA revealed that vetkoek was associated together with mageu, hot maize/sorghum porridge, and instant maize/sorghum porridge (all versions of traditional-type South African foods) and leftovers in their consumption. Vetkoek is a deep-fried leavened, refined wheat flour dough, often sugar-sweetened, which is a popular traditional-type food in South Africa. Its origins are in the cuisine of the Dutch settlers in the Cape who consumed the similar oliebollen [40]. Vetkoek is high in carbohydrates and fat and has a very high energy content, approx. 1029 kJ/100 g [41]. As such, vetkoek and consumption of conventional bread, which was also introduced by European settlers, can be considered as a manifestation of the well-advanced nutrition transition that has been taking place in South Africa [18,19].

Vetkoek was frequently consumed by approximately 24% of the respondents but was far less frequently consumed than bread (69%). It was predominantly a frequent breakfast food of Black consumers (30%). Thus, even today (2022) Black consumers in South Africa tend to follow a combination of traditional and Western eating patterns, as was the case 20 years previous [42]. Also, notably, there was significantly higher consumption by the lower LSM group.

The differences in consumption across racial groups were much smaller for bread, but it was similarly more consumed by the lower LSM group. Significant in these respects was that vetkoek was the food that most reminded respondents of what they grew up with and ranked high as a food that they identified with their culture and tradition. Bread scored far lower in respect to culture and tradition. This indicates that nutrition transition is a complex phenomenon as both vetkoek and bread were historically foods of White people. Notably, vetkoek was one of the foods most consumed with children, whereas bread was mostly consumed by people on their own. These findings concerning tradition and family consumption mirror focus group research which found that among South African consumers across the socioeconomic spectrum, traditional foods were frequently mentioned in connection with nostalgic thoughts of grandmothers [43].

Vetkoek also featured strongly in response to the question “which of these statements best describes what you most want to obtain from the breakfast when you eat?”. It ranked highest regarding being tasty and providing good value for money and joint highest with leftovers regarding “keeping me full longer”. In contrast, vetkoek scored lowest in respect of foods that consumers wanted to make them feel energized/recharged, whereas bread ranked amongst the highest. This is possibly a reflection of the generally high glycemic index of South African breads [44]. However, bread was rated much lower in terms of value for money. In South Africa, as is general in sub-Saharan Africa, the high cost of bread is a major consumer concern [45]. These findings are in general agreement with those in other South African studies [43] where economic factors dominated the discussion of food purchase by lower-income consumers. Also, among low-income consumers, satiety was a more important consideration than among those of higher socioeconomic status.

Concerning convenience, vetkoek and bread were both rated very high as foods that can be consumed quickly. Vetkoek is invariably prepared and sold by street vendors, often at or near minibus taxi ranks and bus stations [46] and not prepared at home, thus, as with bread, requiring minimal meal preparation time.

With respect to consumers wanting breakfast foods that are nutritious/healthy, vetkoek ranked the lowest by far, and bread ranked only 19th out of the 21 foods. Further, as indicated, vetkoek consumption was strongly associated with consumer health concerns about all the issues listed in the questionnaire. This suggests a clear recognition by these consumers of the potential nutritional limitations and the adverse health implications of regular consumption of foods such as vetkoek that are high in carbohydrates and fat but lacking other nutrients [47,48].

#### 4.1.2. Cereals Deep Dive—Functional and High-Fibre Types Compared to Regular RTE Cereals

PCA revealed that all RTE breakfast cereal-types (regular, high-fibre, and functional) were associated together with respect to their consumption. Bran-rich, i.e., high-fibre, cereals date back to 1915–1916 and were developed to address gut health issues, specifically constipation [49]. Functional cereals appeared in the 1970s but gained wider exposure somewhat later [50]. This followed the development of the functional foods concept, foods which provide health benefits beyond basic nutrition [51]. Functional cereals are characterized by containing high levels of nutrients such as protein, dietary fibre, vitamins and essential minerals, and phytochemicals. This contrasts with the relatively high sugar content of certain RTE cereals, particularly those tailored towards children [52]. However, it is notable that some “regular” RTE cereals have similar levels of micronutrients as some “functional cereals”, making distinctions between the two cereal types somewhat opaque.

Functional cereals were consumed regularly by 31.5% of the respondents. High-fibre cereals were even more popular (46% of respondents), which was similar to regular RTE cereals (49.2%). A similar figure, over 50%, for “cereal” (types not distinguished) consumption was found in the UK study of adult breakfast consumption [16]. This present study revealed that older respondents were significantly more likely to consume functional cereals. However, strangely, the proportion consuming high-fibre cereals was generally not higher than the other age groups. In view of the age association with functional cereal consumption, not surprisingly, they were substantially more frequently consumed by people on their own and less frequently with children, and vice versa for regular RTE cereals. Also not surprisingly, a substantially higher proportion of people who had tried two or more diets in the past year were regular consumers of functional- and high-fibre cereals.

Concerning how people wanted to feel when eating breakfast foods, all the cereal types scored high with respect to feeling energized/recharged and a fresh start, and functional cereals scored highest of all the breakfast foods with respect to the respondents wanting the highest level of performance/success. This suggests that the respondents believed that “functional” cereals were truly functional beyond just health enhancement. Regarding what the respondents wanted to obtain when eating, high-fibre cereals scored lowest of all the breakfast foods for “tasty”, despite being so commonly consumed. This indicates that people consume them for health reasons such as improved gut health, which is a well-described benefit of high fibre consumption [53]. The only health concern strongly associated with consumption of functional cereals was poor immunity. This association should be seen in the context of the very high burden of HIV in South Africa [54].

#### 4.1.3. Porridges Deep Dive—Instant Types Compared to Traditional Cooking Types

PCA showed that instant (just mix with hot water and/or milk) oat porridge and instant maize/sorghum porridge both associated closely with their traditional counterparts with respect to consumption. There was little difference in the frequency of common consumption between the instant and traditional porridges, ranging between 36% of the consumers for oat porridge and 29.8% for instant oats. All were substantially less commonly consumed than RTE cereals, which are even more convenient to prepare, i.e., simply adding cold milk. Lower LSM respondents reported significantly higher consumption of traditional cooking porridges, and there was higher consumption in households with children.

Concerning attitudes, all porridges, whether instant or traditional, were strongly associated with respondents wanting to feel energized/recharged and having a fresh start, with traditional cooking porridges being somewhat less so with wanting a fresh start. In this regard, cooking porridges were most strongly associated with their consumption being a habit, reminding them of something they grew up with and identifying with culture/tradition. Notably, oat porridge and muesli were considered as being the most nutritious/healthy breakfast foods. This agrees with our related research which showed that oat porridge was among the foods that these respondents identified as being “whole grains”, and, in turn, they considered that “healthy” was the most important attribute of whole grains [29]. Interestingly, instant maize/sorghum porridge was strongly associated with being tasty. This is likely because many of the products are artificially flavoured and coloured and sugar-sweetened.

Regarding the attribute of convenience, instant maize/sorghum porridge and instant oats tended to be consumed mostly together with children and by people on their own, and “can be consumed quickly” was an attribute associated with instant-type porridges. These suggest that the convenience provided by instant porridges is a consideration. However, there was no positive association between any of the breakfast foods and the statement “Convenience is the most important consideration in my breakfast choices”. Drivers of the similar levels of consumption of maize/sorghum porridge seem to be habit, culture/tradition, and low cost, and for oat porridge, its strong association was with health. These findings are in general agreement with a study of the drivers of food purchases by South African consumers [43]. Convenience was found to be more important to middle- and high-income consumers, whereas economic considerations were the predominant driver with lower-income consumers. Further, lower- and middle-income consumers expressed the opinion that traditional foods are healthier than modern foods, which suggests that these consumers may have an innate negative attitude to instant versions of traditional porridges regardless of their nutritional content or product claims.

### 4.2. Public Health Recommendations

The challenge is how these somewhat conflicting consumer needs of better nutrition and improved wellness versus cost, convenience, taste, and satiety can be met in developing economy countries such as South Africa. To achieve this, a trans-disciplinary approach is required, where food technologists, nutritionists, public health scientists, and consumer scientists work in unison. Pre-cooked (ready-to-consume) foods and beverages based on blends of whole-grain cereals and pulses are a good prospect as they are least-costly nutrient-dense foodstuffs [55,56]. Data from the same survey was previously used by the present authors to provide a deeper understanding of whole grain knowledge, attitudes, and behaviour [29], and in this context the importance of the breakfast-eating occasion in supporting whole grain intake cannot be understated given the importance of bread, hot cereals, and RTE cereals to whole grain intakes [57].

The critical question is how the results of this study can be used to inform dietary recommendations, policy, and communication to help improve the population’s diet. Specifically, it was observed that breakfast may be perceived more than other meals as central to health and nutrition, which presents the opportunity to leverage this eating occasion towards improving diet. Furthermore, it indicates that most breakfast foods are consumed at home, suggesting that policies focused on at-home food consumption are more likely to have a major impact on consumers to make healthy breakfast food choices. This highlights the need for dietary recommendations and advice to be context-specific and based on the real-world eating habits of the general population. Consumer education around the importance of breakfast and specific foods that ought to be consumed is therefore a likely avenue of action that could be pursued. The data collected here can be leveraged by NGOs and consumer advocacy groups to inform such campaigns and efforts. It is also important to keep in mind how other public health policy areas may interact with breakfast food choices, including issues related to mandatory or voluntary front-of-pack labelling schemes, whole grain labelling, and food subsidies/taxation schemes.

### 4.3. Study Strengths and Limitations

This study has several noteworthy strengths and limitations. First, the design of the questionnaire enabled exploration of associations between the types of foods that people consume for breakfast and a wide range of factors that could influence their consumption, including sociodemographic and consumer behaviour, needs, desires, and concerns. This is particularly important in developing-economy countries where there is a lack of timely nationally representative dietary data. While online surveys do not generate the same quality of data as highly controlled scientific surveys because, for example, they may introduce more recall bias than face-to-face interviews, they can fill important gaps in understanding critical dietary behaviours. Also, although the sample size was relatively small, due to it being balanced with respect to South Africa’s demographics, it revealed numerous valuable sub-group differences by gender, age group or ethnicity. Among the study limitations, only individuals of moderate and higher socioeconomic status were sampled, which means the results are not generalizable to the entire South African adult population. Individuals who did not frequently consume breakfast were also not surveyed. As there were some data quality concerns regarding the number of breakfast food types reported by participants, it was decided based on international data to remove those respondents reporting frequent consumption of five or more foods. Lastly, while cross-sectional data can provide valuable insights into current patterns of behaviour and motivations, such data do need to be interpreted cautiously, and no causal claims should be made regarding any of the observed associations.

Despite limitations, this study indicates that South Africans may consider breakfast foods differently than other components of their diet, placing a somewhat higher emphasis on nutrition and well-being than simple cost. These findings suggest that the opportunity exists to assist consumers in developing-economy countries in making breakfast food choices that support their health and well-being.

## 5. Conclusions

This study has confirmed the hypothesis that even among persons of moderate to higher living standard, a wide variety of breakfast foods are consumed by South African adults, ranging from traditional-type African foods such as vetkoek and mageu to modern “Western” foods like functional breakfast cereals and shakes. It has also confirmed that the drivers of breakfast food choice in South Africa are very complex and that this complexity seems to be related to its population being economically, ethnically, and culturally very diverse. The main drivers of their food choices were also diverse, including nutrition and health, taste, value for money, and the desire to feel energized/recharged. Although convenience was not a strong overt driver, it was reflected in the desire for breakfast foods that are quick to consume. Thus, a simple hierarchy of choice is unlikely to explain why South African consumers make their choices.

Notwithstanding that the study was limited to those of moderate and higher income, it indicates that beyond current breakfast food consumption behaviour, South African consumers have needs that cut across population groups, cultures, and economic status. Notably, perceived healthfulness and nutritional value were amongst the most reported benefit areas and were the top reported benefit area for 13 of the 21 assessed breakfast food types. Additionally, various aspects of wellness, for example, feeling energized/recharged, were very highly rated. That South African consumers have these needs indicates that they can be influenced to make more healthy breakfast choices through effective social and behaviour communications. To help ensure that these communications are successful, studies using projective techniques, including storytelling, picture interpretation, and third-person techniques, should be considered to gain a deeper understanding of consumers’ covert motivations.

## Figures and Tables

**Table 1:** Respondent sample demographical characteristics and percent of sample consuming breakfast every day.

	N	% of Sample	Eating Breakfast Daily, %
Total	842	100.0	72.3
Age group, y			
18–24	149	17.7	67.8
25–34	264	31.4	68.9
35–44	200	23.8	73.0
45–54	141	16.8	75.9
55–64	88	10.5	83.0
Gender			
Female	435	51.7	69.7
Male	407	48.3	75.2
Living Standard Measure (LSM)			
5–7	496	58.9	75.6
7–10	346	41.1	67.6
Population group (Race)			
Black	637	75.7	74.6
Coloured/Indian	109	13.0	69.7
White	96	11.4	60.4
Children in household			
Yes	656	77.9	72.7
No	186	22.1	71.0
Self-rated health			
Excellent/very good	359	42.6	78.3
Good	363	43.1	70.3
Fair to poor	120	14.3	60.8

**Table 2:** ANOVA tests of between-participant effects—breakfast food categories by independent variables.

Univariate ANOVA 1—Dependent Variable: “On-the-Go” Food Category					
Independent Variables	Type III SS	df	Mean Square	F	Sig.
Corrected Model	197,372 ^a1^	10	19.737	21.376	<0.001
Intercept	204.503	1	204.503	221.481	<0.001
Population group (Race)	12.178	2	6.089	6.595	0.001
Gender	2.592	1	2.592	2.807	0.094
LSM (Living standard)	5.441	1	5.441	5.893	0.015
Perceived health status	52.558	2	26.279	28.461	<0.001
Concern about hypertension and blood pressure	20.517	2	10.258	11.110	<0.001
Taste is more important in breakfast than health and wellness	24.717	1	24.717	26.769	<0.001
I actively look for whole grain foods for my breakfast occasions	24.490	1	24.490	26.524	<0.001
Error	767.298	831	0.923		
Total	7526.250	842			
Corrected Total	964.670	841			
Univariate ANOVA 2—Dependent variable: “Traditional” food category					
Independent variables	Type III SS	df	Mean Square	F	Sig.
Corrected Model	174,065 ^a2^	11	15.824	17.465	<0.001
Intercept	1757.051	1	1757.051	1939.205	<0.001
Age	14.509	4	3.627	4.003	0.003
Population Group (Race)	40.526	2	20.263	22.364	<0.001
LSM (Living standard)	6.634	1	6.634	7.322	0.007
Perceived health status	26.191	2	13.096	14.453	<0.001
Concern about hypertension and blood pressure	40.289	2	20.144	22.233	<0.001
Error	752.036	830	0.906		
Total	6312.320	842			
Corrected Total	926.101	841			
Univariate ANOVA 3—Dependent variable: “RTE and functional cereals” food category					
Independent variables	Type III SS	df	Mean Square	F	Sig.
Corrected Model	96,299 ^a3^	6	16.050	16.288	<0.001
Intercept	431.646	1	431.646	438.055	<0.001
Population group (Race)	12.296	2	6.148	6.239	0.002
LSM (Living standard)	11.318	1	11.318	11.486	<0.001
Perceived health status	27.410	2	13.705	13.908	<0.001
I actively look for whole grain foods for my breakfast occasions	29.215	1	29.215	29.648	<0.001
Error	822.784	835	0.985		
Total	7908.960	842			
Corrected Total	919.084	841			

SS = Sum of squares. ^a1^. R Squared = 0.205 (Adjusted R Squared = 0.195); ^a2^. R Squared = 0.188 (Adjusted R Squared = 0.177); ^a3^. R Squared = 0.105 (Adjusted R Squared = 0.098). Covariates in the models were evaluated at the following mean values: “Taste is more important in breakfast than health and wellness” (mean = 4.87); “I actively look for whole grain foods my breakfast occasions” (mean = 6.09).

## Data Availability

The de-identified data is available on request.

## Supplementary Materials

The following supporting information can be downloaded at: https://www.mdpi.com/article/10.3390/foods15010014/s1, The questionnaire questions and response options.
